# Supplementary material for: More frequent extreme climate events stabilize reindeer population dynamics
Source: Nat Commun. 2019 Apr 8;10:1616. doi: 10.1038/s41467-019-09332-5 (PMC6453938; doi:10.1038/s41467-019-09332-5)
Supplement: Supplementary file 1 — Supplementary Information [file 41467_2019_9332_MOESM1_ESM.pdf]

Supplementary Information

More frequent extreme climate events stabilize reindeer population dynamics

Hansen, Gamelon, et al.

Supplementary Figure 1 | Annual estimated (black lines) and 'observed' (i.e. mean of 9,090 posterior samples from the IPM, red lines) survival rates for the six age classes of female Svalbard reindeer. Estimations are from a 'step-by-step' approach, i.e. each year is estimated based on the 'observed' age-specific population sizes, amount of rain-on-snow (ROS) and winter length. **a**, Age class 1 = calves. **b**, Age class 2 = yearlings. **c**, Age class 3 = two-year. **d**, Age class 4 = 3-8 year. **e**, Age class 5 = 9-11 years. **f**, Age class 6 = 12 years and older.

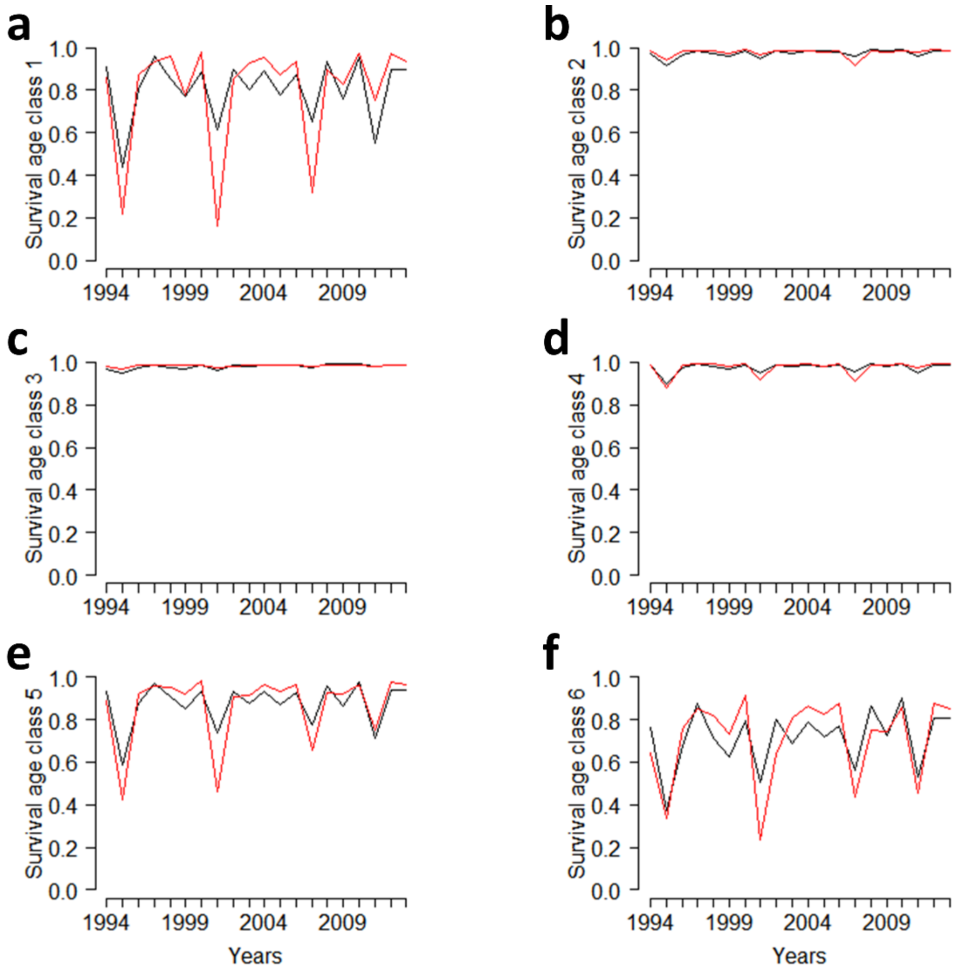

Supplementary Figure 2 | Annual estimated (black lines) and 'observed' (i.e. mean of 9,090 posterior samples from the IPM, red lines) fecundity rates for five age classes (calves, i.e. age class 1, do not get pregnant) of female Svalbard reindeer. Estimations are from a 'step-by-step' approach, i.e. each year is estimated based on the observed age-specific population sizes the previous year, amount of rain-on-snow (ROS) and winter length. **a**, Age class 2 = yearlings. **b**, Age class 3 = two-year. **c**, Age class 4 = 3-8 year. **d**, Age class 5 = 9-11 years. **e**, Age class 6 = 12 years and older.

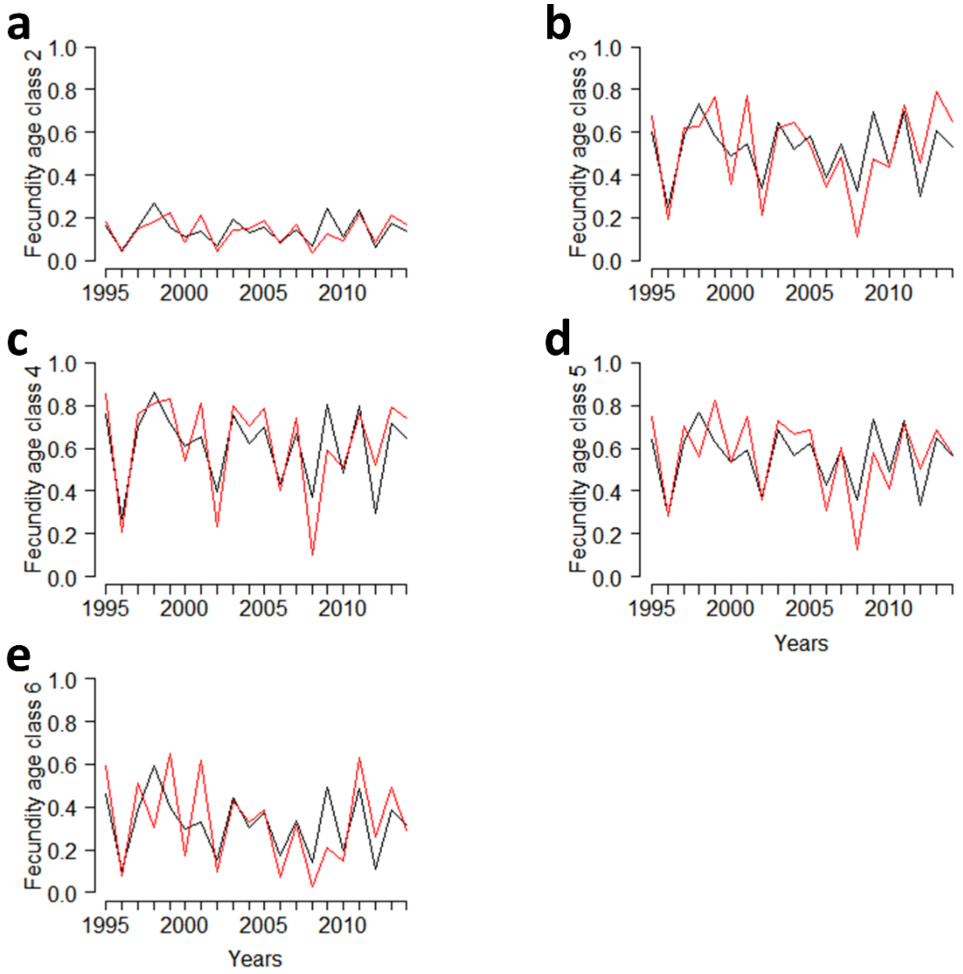

Supplementary Figure 3 | Fluctuations in age structure of female Svalbard reindeer during the study period (i.e. mean of 9,090 posterior samples from the IPM), shown as the accumulated proportion of reindeer with increasing age. Age_0 = calves, Age_1 = yearlings, Age_2 = two-year, Age_3.8 = 3-8 year, Age_9.11 = 9-11 years, Age_12 = 12 years and older.

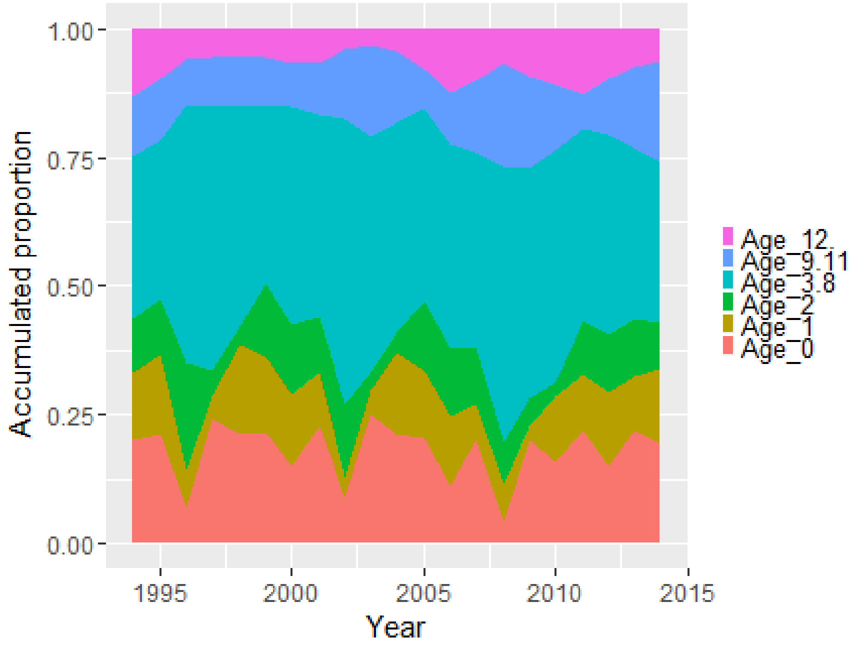

Supplementary Figure 4 | Annual estimated (black symbols) and 'observed' (i.e. from IPM) total population sizes of female Svalbard reindeer. Estimations are from a 'step-by-step' approach, i.e. each year's population size is estimated based on the 'observed' age-specific population sizes and weather the previous year, and the corresponding estimated survival and fecundity rates (see Supplementary Figures 1 and 2). Error bars represent one standard error.

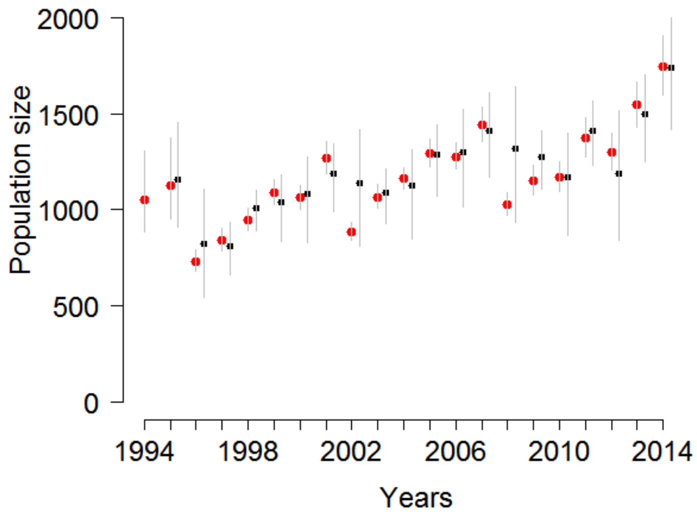

Supplementary Figure 5 | Comparison of 'observed' and estimated population growth rates of female Svalbard reindeer. a, Annual estimated (black lines) and 'observed' (i.e. mean of posterior samples from IPM, red lines) population growth rates. Estimations are from a 'step-by-step' approach, i.e. each year's population growth rate is estimated based on the 'observed' age-specific population sizes and weather the previous year. **b**, Estimated population growth rates plotted against 'observed' population growth rates. The 1:1 relationship is shown for illustration.

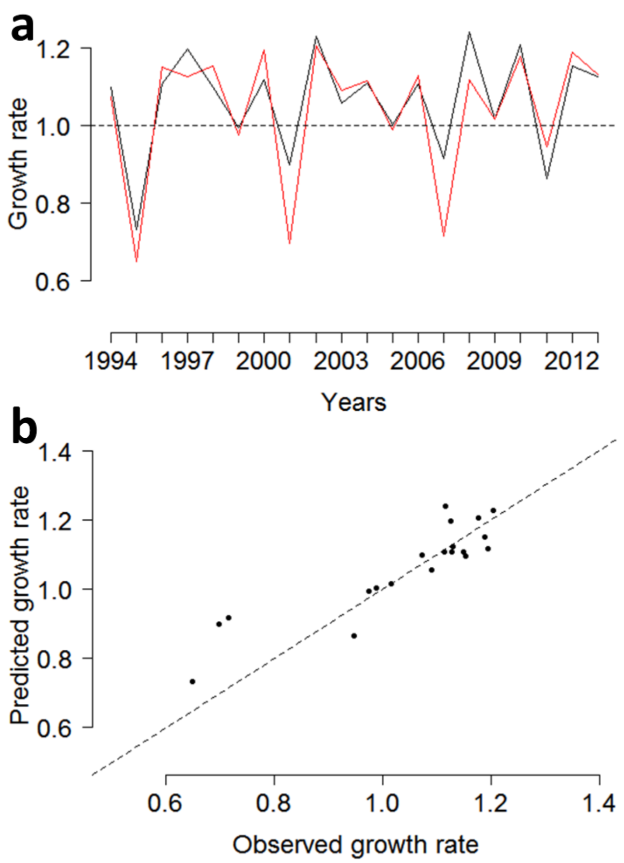

Supplementary Figure 6 | Simulations of five different rain-on-snow (annual winter rain amount, mm) scenarios. a, The cumulative distribution function for observed values of rain amount (1962-2014, black dashed line) as well as the cumulative distribution function after applying the beta distribution transformation for different scenarios. The ‘medium’ scenario simulates the historical state. **b**, The resulting (smoothed) probability densities associated with the different scenarios. **c**, Example time series simulation of rain-on-snow for each scenario.

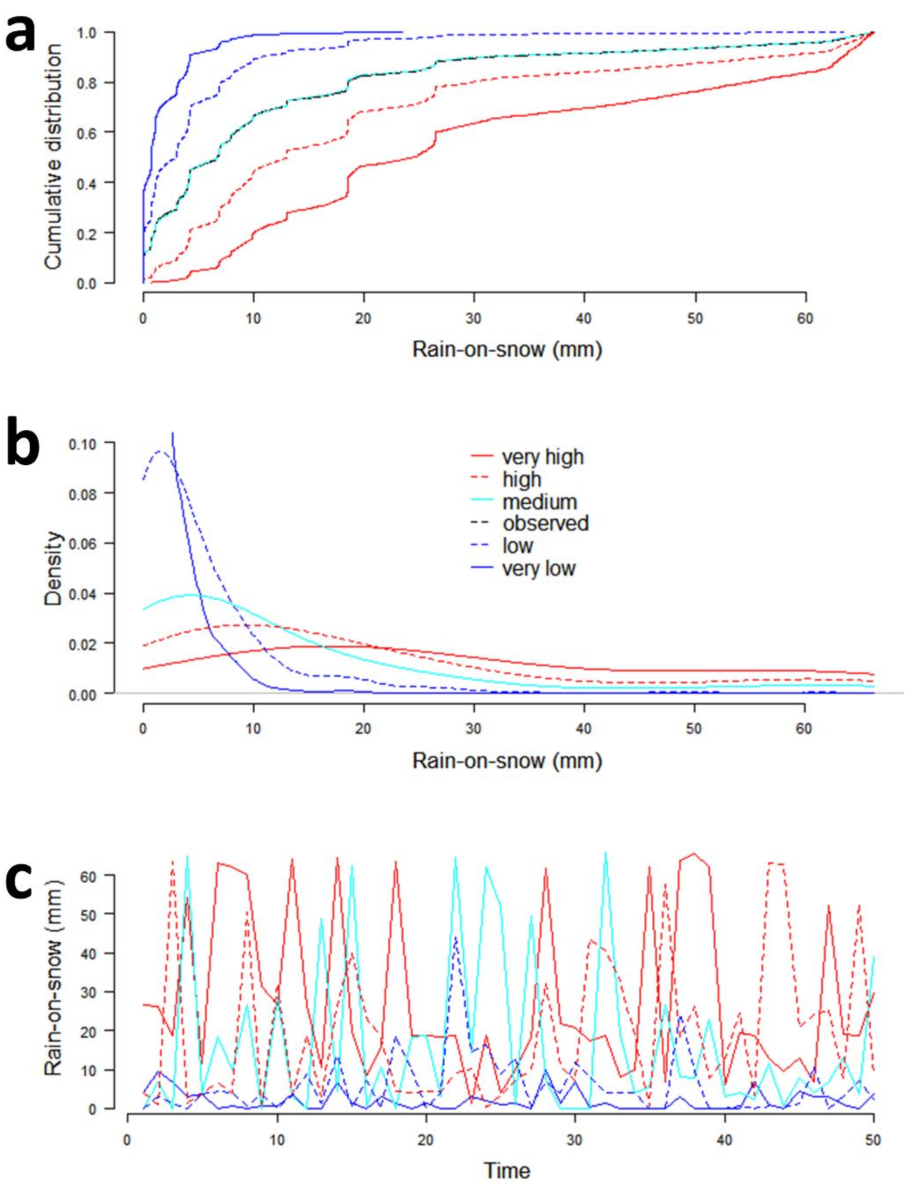

Supplementary Figure 7 | The importance of the time elapsed between two 'extreme ROS winters' for the age structure fluctuations in female Svalbard reindeer. Time-series of proportion prime-aged females (i.e. age class 4 [3-8 years of age]) based on deterministic simulations with a high initial population size (N = 1700) and low initial proportion of prime-aged individuals (28%) (see Fig. 6 a for corresponding population sizes). ROS scenarios differ from left to right by an increasing time elapsed from the first extreme ROS winter in year $t = 0$ (shown by red vertical line) to the second extreme ROS winter (shown by blue vertical line). Note that in the leftmost panel, there is no second extreme ROS winter, and the population approaches stable age structure over time. An extreme ROS winter is modelled as the observed maximum value (i.e. in winter 1996), while all other years are kept at the observed mean during the reindeer data time-series (1994-2014).

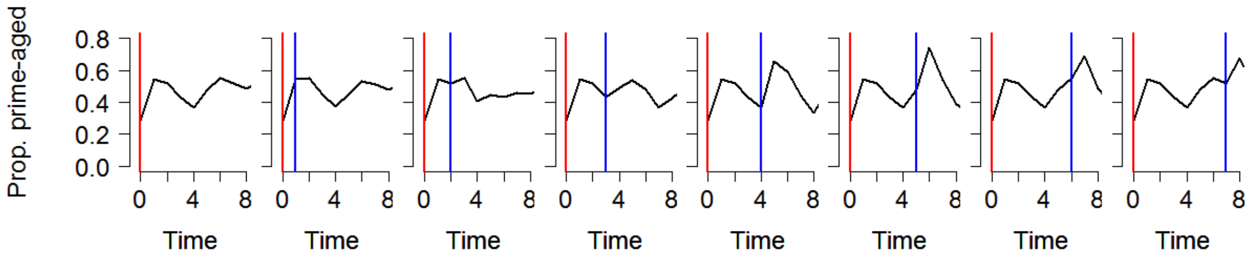

Supplementary Figure 8 | The density-dependent rain-on-snow (ROS) effect on female Svalbard reindeer survival. For 100 random samples out of the 9,090 posterior samples ('population models'), the density effect is shown for two extreme values of rain-on-snow (0 mm [blue curves] and 66 mm [red curves]) and for different estimated values of *k* in the density dependent (ROS) effect ($ROS' = ROS_t \times e^{k \times N_{posthunt,t}}$).

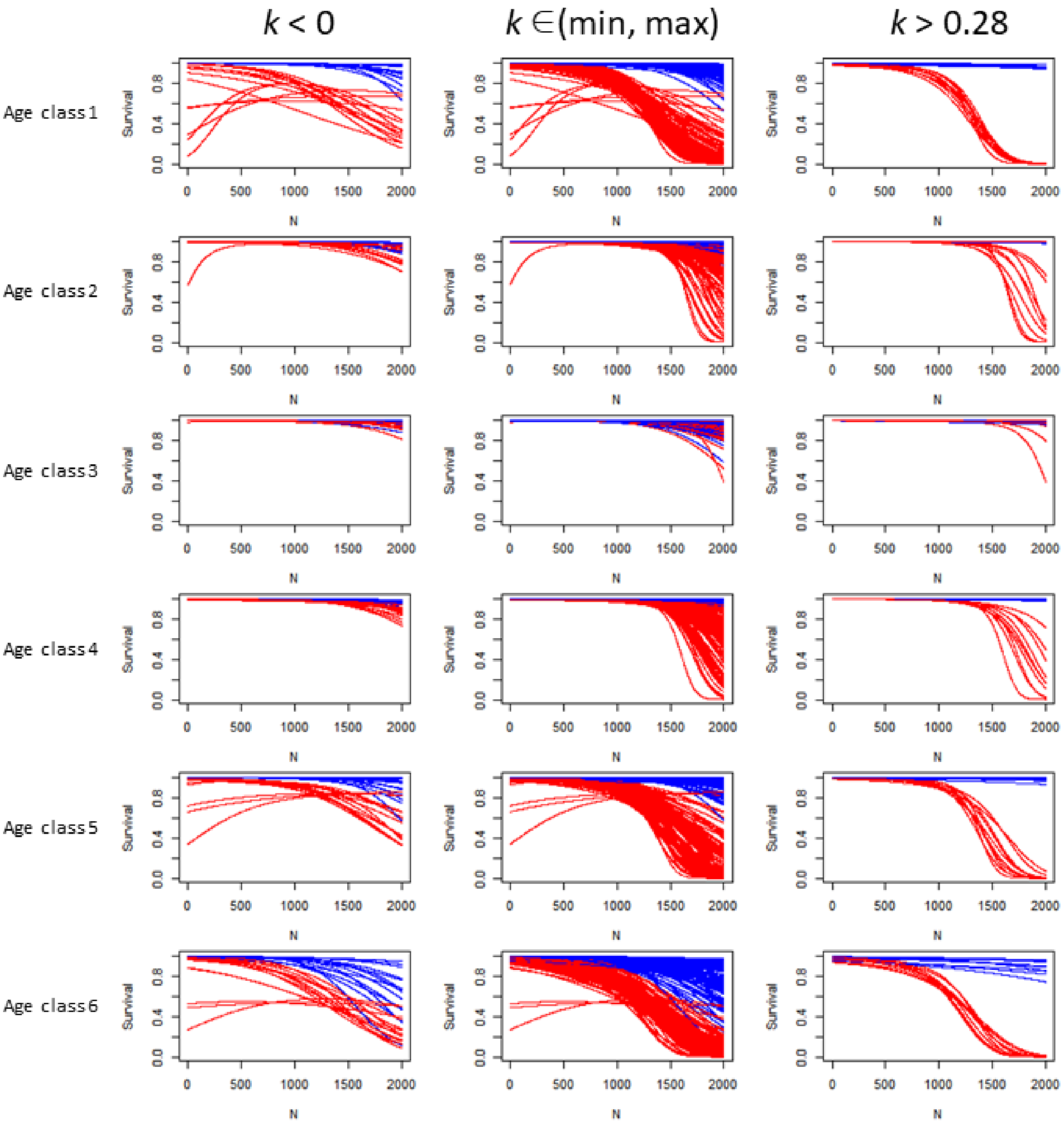

Supplementary Figure 9 | The density-dependent rain-on-snow (ROS) effect on female Svalbard reindeer fecundity. For 100 random samples out of the 9,090 posterior samples ('population models'), the density effect is shown for two extreme values of rain-on-snow (0 mm [blue curves] and 66 mm [red curves]) and for different estimated values of k in the density dependent (ROS) effect ($ROS' = ROS_t \times e^{k \times N_{posthunt,t}}$).

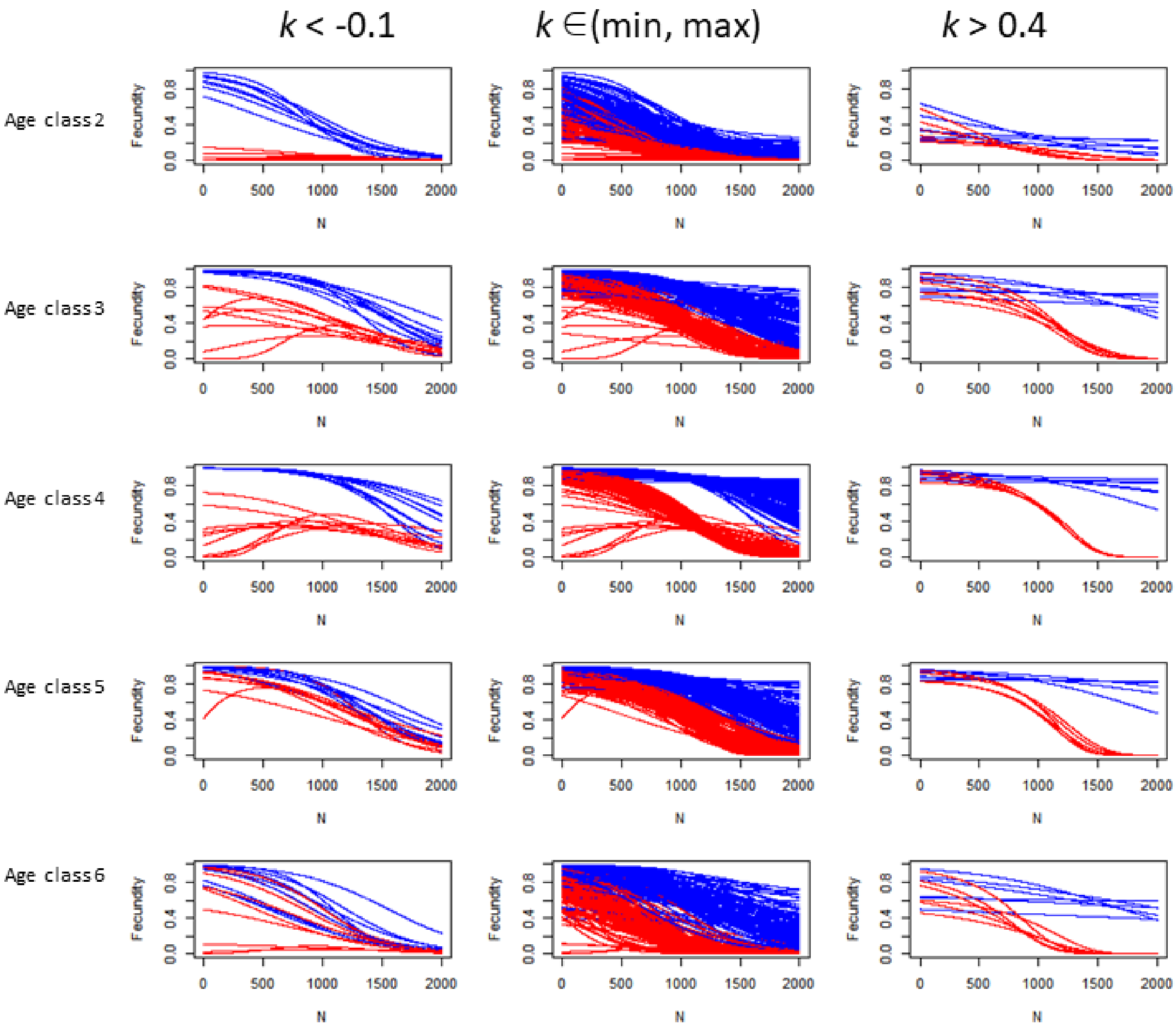

Supplementary Table 1 | Climate-density effects on population growth rates in wild Svalbard reindeer. Parameter estimates and their associated standard error (SE) and P-value are reported from linear regression models of the effects of population size (detrended and log-transformed, *N*), rain-on-snow (ROS), winter length (days), and the interaction of population size and ROS, on annual population growth rate of the study population between 1994 and 2014. The analysis was first performed using population size *N* as a continuous variable and then, for illustrative purpose (Fig. 1 c), by categorizing *N* into three levels of population densities (low, medium, high).

Parameter	Estimate (SE)	P-value
**Using population size *N***		
Intercept	1.14 (0.45)	0.02
N	-0.03 (0.34)	0.93
ROS	-0.07 (0.02)	<0.01
Winter length	-4.10^{-3} (2.10^{-3})	0.05
N:ROS	-0.38 (0.13)	0.01
**Using three levels of population densities: Low, Medium, High**		
Intercept	0.92 (0.50)	0.09
ROS	-0.14 (0.03)	<0.01
N Low	5.10^{-3} (0.11)	0.97
N Medium	0.11 (0.13)	0.43
Winter length	-3.10^{-3} (2.10^{-3})	0.15
N Low:ROS	0.11 (0.04)	0.03
N Medium:ROS	0.05 (0.05)	0.35

Supplementary Table 2 | Climate-density effects on survival and fecundity in wild Svalbard reindeer. Mean parameter estimates from linear mixed effects models on each of 9,090 posterior samples (credible intervals in brackets) are reported for the effects of age class, year (i.e. trend effect, centered), winter length, population size ($N_{posthunt}$, standardized), and a density-dependent, age-specific rain-on-snow (ROS) effect ($ROS' = ROS_t \times e^{k \times N_{posthunt,t}}$), on survival S_i (logit-transformed) and fecundity rates F_i (logit-transformed) of female reindeer of age class i, between 1994 and 2014.

Parameter	Mean posterior sample
**Survival**	
Age class 1	11.51 [6.81; 17.52]
Age class 2	12.81 [7.39; 20.17]
Age class 3	12.63 [7.39; 19.38]
Age class 4	13.41 [8.44; 19.77]
Age class 5	11.87 [6.84; 18.00]
Age class 6	10.01 [5.21; 15.98]
$N_{posthunt}$	-0.49 [-0.89; -0.10]
year	0.10 [0.05; 0.16]
Winter length	-0.03 [-0.06; -0.01]
ROS' Age class 1	-0.81 [-1.07; -0.57]
ROS' Age class 2	-0.38 [-1.07; 0.02]
ROS' Age class 3	-0.19 [-0.74; 0.20]
ROS' Age class 4	-0.58 [-1.00; -0.24]
ROS' Age class 5	-0.71 [-1.17; -0.35]
ROS' Age class 6	-0.53 [-0.88; -0.19]
k	0.13 [-0.08; 0.36]
**Fecundity**	
Age class 2	0.54 [-2.90; 3.76]
Age class 3	2.85 [-0.66; 6.09]
Age class 4	3.69 [0.16; 6.89]
Age class 5	3.02 [-0.61; 6.18]
Age class 6	2.18 [-1.62; 5.65]
$N_{posthunt}$	-0.42 [-0.86; -0.01]
year	0.07 [0.02; 0.13]
Winter length	-0.01 [-0.02; 0.01]
ROS' Age class 2	-0.38 [-0.79; -0.08]
ROS' Age class 3	-0.43 [-0.71; -0.19]
ROS' Age class 4	-0.59 [-0.77; -0.41]
ROS' Age class 5	-0.42 [-0.71; -0.11]
ROS' Age class 6	-0.68 [-1.33; -0.02]
k	0.15 [-0.23; 0.48]
